# Acupuncture combined with transcutaneous auricular vagus nerve stimulation for chronic postpartum insomnia comorbid with emotional disorders: a case report

**DOI:** 10.3389/fmed.2026.1918028

**Published:** 2026-10-02

**Authors:** Qin Lyu, Re-Yi-Han-Gu-Li Ju-Lai-Ti, Shu-Min Wang, Miao-Miao Jing, Yi-Fei Li, E. Zhou, Tian-Yu Bai

**Affiliations:** 1 Department of Acupuncture-Moxibustion and Tuina, Shandong Provincial Third Hospital, Shandong University, Jinan, Shandong, China; 2 Department of Traditional Chinese Medicine, Uyghur Medicine Hospital of Kashgar Prefecture, Kashgar, Xinjiang Uygur Autonomous Region, China; 3 Graduate School, Shandong Sport University, Jinan, Shandong, China

**Keywords:** acupuncture, anxiety, chronic insomnia, depression, transcutaneous auricular vagus nerve stimulation

## Abstract

Postpartum women constitute a vulnerable population in whom insomnia comorbid with emotional disorders such as anxiety and depression is disproportionately prevalent, attributable to hormonal fluctuations, environmental changes, and social role transitions. This comorbidity significantly increases their long-term risk of mental health disorders. Given the safety concerns of sedative-hypnotic medications, non-pharmacological therapies are the first-line treatment for this population. We report the case of a 34-year-old woman with chronic postpartum insomnia comorbid with emotional disorders, who provided written informed consent for the publication of her de-identified clinical data and received 4 weeks of combined acupuncture and transcutaneous auricular vagus nerve stimulation (taVNS) treatment. Clinical assessments were performed at baseline, week 2, week 4 (end of treatment), and week 8 (4-week post-treatment follow-up) using validated scales including the PSQI, ISI, and HAMA, among others. After 4 weeks of treatment, the patient demonstrated significant improvements in objective sleep quality and anxiety symptoms, while subjective insomnia perception and depressive symptoms remained partially unresolved. Notably, all clinical outcomes continued to improve to near-normal levels during the follow-up period without any additional intervention, and no adverse events were recorded. This case demonstrates that combined acupuncture and taVNS is a safe and effective intervention for chronic postpartum insomnia comorbid with emotional disorders, with a favorable sustained therapeutic effect after treatment cessation, offering a valuable alternative for non-pharmacological management in this population.

## Highlights

- Four-week combined acupuncture and taVNS improved sleep, emotional symptoms and quality of life in a patient with chronic postpartum insomnia comorbid with emotional disorders.
- The acupuncture prescription was formulated based on TCM syndromes of heart-kidney disharmony and heart-spleen deficiency.
- taVNS exerts synergistic effects with acupuncture by modulating autonomic nervous system function.
- Therapeutic effects were sustained and further enhanced during the 4-week treatment-free follow-up after treatment cessation.

## 1 Introduction

Sleep disorders are among the most prevalent worldwide. Chronic insomnia disorder, defined by the International Classification of Sleep Disorders, 3rd edition (ICSD-3), is characterized by sleep disturbances occurring at least three nights per week for a minimum of three months, with sleep-onset latency >30 min, ≥2 nocturnal awakenings, early-morning awakening, or total sleep time <6.5 h, accompanied by daytime fatigue, impaired concentration, and functional impairment (1, 2). Up to 10% of adults meet criteria for chronic insomnia disorder (2). Women are disproportionately affected, with a prevalence of 12.8% compared with 9.7% in men (3).

The perinatal period is not only a critical window for the onset of insomnia, but also associated with a significantly elevated risk of depression, anxiety, and even suicide-related outcomes among women (4). Reported prevalence of perinatal insomnia varies substantially depending on assessment tools, sampling populations, and diagnostic criteria, with Western cohorts reporting approximately 40%–60% and Asian cohorts approximately 30%–50%, though the proportion of severe or persistent insomnia remains relatively stable (5, 6). Postpartum hormonal shifts, disrupted circadian rhythms, infant care demands, and role transition frequently trigger or exacerbate sleep disturbances (4). A substantial proportion of women continue to experience insomnia symptoms beyond 2 years postpartum, indicating that postpartum insomnia is not merely a transient adjustment phenomenon but a distinct clinical entity with a chronic trajectory (7, 8).

From a diagnostic perspective, when insomnia symptoms begin during the postpartum period but persist beyond the typical perinatal window, this condition should be classified as chronic insomnia disorder with postpartum onset rather than “postpartum insomnia”. A term typically reserved for transitional sleep disturbance arising within the first postpartum year (9). Untreated postpartum insomnia increases the risk of clinically significant anxiety, depression, and cardiovascular strain (10).

Although cognitive behavioral therapy for insomnia (CBT-I) is the first-line standard (11, 12), many postpartum women struggle with fragmented schedules and inconsistent implementation of sleep restriction and stimulus control strategies. Thus, flexible, easily integrated non-pharmacological interventions are urgently needed. Acupuncture has been validated for insomnia (13) by modulating *γ*-aminobutyric acid (GABA), melatonin, and hypothalamic-pituitary-adrenal (HPA) axis activity to suppress hyperarousal (14). Specifically, acupuncture downregulates HPA axis hyperactivity, reducing excessive secretion of corticotropin-releasing hormone (CRH) and cortisol that directly inhibit sleep-promoting neurons in the ventrolateral preoptic nucleus (VLPO). It upregulates GABAergic inhibitory neurotransmission, restoring thalamocortical sleep spindle regulation. It stabilizes the circadian rhythm of melatonin secretion, thereby facilitating sleep initiation and maintenance (15, 16).

Over the past decade, taVNS has emerged as a novel neuromodulatory technique for sleep disorders (17, 18). The cymba conchae is the exclusive cutaneous distribution area of the auricular branch of the vagus nerve. Its stimulation activates the nucleus tractus solitarius, enhances parasympathetic output, reduces sympathetic tone, and improves heart rate variability and baroreflex sensitivity, creating favorable neurovisceral conditions for sleep initiation and maintenance (19). Preliminary studies indicate that taVNS enhances sleep quality and duration in primary insomnia by modulating functional connectivity within the default mode network and between the default mode network and other brain networks (20). The two modalities target completely independent yet complementary neurobiological pathways. Acupuncture primarily engages central sleep–emotion circuitry, while taVNS corrects peripheral sympathovagal imbalance, forming a “central–peripheral” synergistic regulatory framework. However, detailed protocols and clinical evidence for combined acupuncture and taVNS in chronic postpartum insomnia remain limited, especially in patients with prominent autonomic dysfunction and severe anxiety. Direct evidence of synergistic effects is currently lacking, as the complementarity can only be inferred from their respective independent pathways at present.

This case report describes a 34-year-old woman with chronic postpartum insomnia comorbid with anxiety and depression, presenting with sleep-onset difficulty and light sleep, who was treated with combined acupuncture and taVNS. We detail the implementation, challenges, and clinical outcomes of this combined regimen in this unique life-stage population. This report follows the CARE guidelines for case reports.

## 2 Case presentation

### 2.1 Clinical assessment

A 34-year-old female healthcare professional undergoing advanced training alone away from home and without cohabitation with her child presented with a 2-year history of persistent sleep-onset difficulty and light, fragmented sleep. Symptoms began after full-term vaginal delivery: sleep-onset latency of 60-120 min, 1-5 nocturnal awakenings, frequent dreaming, and early-morning awakening at 4:30 AM with inability to return to sleep. She reported marked daytime fatigue, poor concentration, irritability, nervousness, rumination, occasional palpitations, and dry stools. Symptoms worsened in the preceding 6 months due to heavy training workload, shared accommodation, and lack of social support. She had not received standardized insomnia treatment and had never used benzodiazepines or other hypnotic medications.

From a diagnostic standpoint, although the patient's insomnia symptoms began during the postpartum period, the 2-year disease course has extended well beyond the typical perinatal time window. According to the DSM-5 criteria for chronic insomnia disorder and the ICSD-3, this condition should be classified as chronic insomnia disorder with postpartum onset, rather than “postpartum insomnia”, which typically refers to sleep disturbance arising within the first postpartum year and directly related to maternal-infant adaptation (9, 10).

Neurological examination was unremarkable, no hallucinations, delusions, suicidal ideation, obsessive-compulsive symptoms, or panic attacks were detected. Baseline scores were: Pittsburgh Sleep Quality Index (PSQI) 14, Center for Epidemiologic Studies Depression Scale (CES-D) 27, Hospital Anxiety and Depression Scale-Anxiety (HADS-A) 8, Insomnia Severity Index (ISI) 13, Hamilton Anxiety Scale (HAMA) 23, Hamilton Depression Rating Scale-17 (HAMD-17) 9. The patient had no prior psychiatric history, no major medical conditions, and no history of alcohol or substance abuse. She was diagnosed with chronic insomnia disorder per ICSD-3 criteria. The patient provided written informed consent after a detailed explanation of the mechanism, expectations, and potential mild discomfort of combined acupuncture and taVNS, and agreed to the publication of her de-identified clinical data.

From the perspective of Traditional Chinese Medicine (TCM), inspection revealed a red tongue with thin coating and a thin, rapid pulse, suggestive of a primary pattern of heart-kidney disharmony accompanied by concurrent heart-spleen deficiency (21).

In classic TCM theory, the Heart corresponds to “Fire” and the Kidney corresponds to “Water” under physiological homeostasis. Normal yin-yang balance relies on descending Heart-Fire warming Kidney-Water, while ascending Kidney-Water nourishes Heart-Fire-a dynamic equilibrium termed “harmonious interaction between Water and Fire” (22). Postpartum hemorrhage and sustained mental strain are hypothesized to consume Kidney-Yin, which may fail to ascend and restrain upward blazing Heart-Fire, creating a state of heart-kidney disharmony (21). From modern neuroendocrine perspectives, this TCM pattern may partially correspond to impaired neuroendocrine negative feedback, sustained sympathetic overactivity, and flattened melatonin diurnal rhythms; elevated “Heart-Fire” could potentially correlate with chronic HPA axis hyperarousal, a biological state widely observed in insomnia cohorts (22). Such overlapping physiological disturbances may contribute to prolonged sleep onset, frequent disturbing dreams, palpitations, and rumination, though direct causal linkage between this specific TCM tongue-pulse presentation and single hormonal markers remains unconfirmed in large-scale postpartum samples (13).

The Spleen governs the generation and transformation of qi and blood, while the Heart stores blood and houses mental consciousness. Postpartum qi-blood depletion combined with chronic mental exertion may jointly damage splenic transport and heart blood storage, potentially resulting in insufficient heart blood to nourish the spirit. Modern mechanistic studies propose tentative correlations between this heart-spleen deficiency pattern and diminished central GABA ergic inhibitory signaling, irregular diurnal cortisol secretion, and reduced parasympathetic vagal tone, which might collectively explain shallow, easily-interrupted sleep, persistent daytime fatigue, low mood, and irregular bowel function as reported in this patient (23, 24). Notably, these parallel biological changes represent associative hypotheses rather than definitive one-to-one equivalents for TCM deficiency patterns, and most supporting data derive from non-postpartum insomnia populations.

### 2.2 Intervention protocol

#### 2.2.1 Acupuncture protocol

Based on the TCM pathogenesis of heart-kidney disharmony and heart-spleen deficiency, the main points selected were Shenmen (HT7), Shaohai (HT3), Yingu (KI10), and Zusanli (ST36). Acupuncture was performed strictly in accordance with WHO Standard Acupuncture Point Locations by a licensed acupuncturist with 6 years of clinical experience.

(1) Morning Sessions (before noon, yang phase)
  Yang meridian: Left Zusanli (ST36), needle tip directed toward the head
  Yin meridians: Right Shenmen (HT7), needle tip directed toward the head; Right Yingu (KI10), needle tip directed toward the head; Right Shaohai (HT3), needle tip directed toward the hand.
(2) Afternoon Sessions (after noon, yin phase)
  Yang meridian: Right Zusanli (ST36), needle tip directed toward the head
  Yin meridians: Left Shenmen (HT7), needle tip directed toward the head; Left Yingu (KI10), needle tip directed toward the head; Left Shaohai (HT3), needle tip directed toward the hand
(3) Needle Direction and Reinforcing-Reducing Mechanism
  The Heart Meridian of Hand-Shaoyin runs from the chest to the hand. Following the meridian flow is reinforcing; opposing the meridian flow is reducing:
  Shenmen (HT7, at the wrist): Needle directed toward the head (against meridian flow) to reduce heart fire, calm the mind, and tranquilize the spirit
  Shaohai (HT3, at the elbow): Needle directed toward the hand (along meridian flow) to tonify heart blood, nourish heart yin, and lead fire back to its source
  Yingu (KI10) and Zusanli (ST36) were directed toward the head to promote yang ascent, coordinate heart and kidney, and strengthen the spleen.
(4) Procedure Details
  Disposable sterile stainless steel needles (0.25 mm × 25 mm, Hwato, China) were inserted at a 45° oblique angle until deqi (sensation of soreness, numbness, distension) was obtained. Needles were retained for 60 min with manual manipulation once every 20 min. From week 3 onward, Baihui (DU20) and Sishencong (EX-HN1) were added with transverse insertion to enhance sedative and tranquilizing effects.

#### 2.2.2 taVNS protocol

taVNS was delivered using a transcutaneous electrical nerve stimulator (tVNS501, Changzhou Rsma Medical Equipment Co., Ltd., China). Self-adhesive electrodes were applied to the left cymba concha, the region with the highest density of vagus nerve afferents. Stimulation parameters were set as follows: frequency 30 Hz, pulse width 200*μ*s, 30-second on/30-second off duty cycle. The intensity was titrated from 0.5 mA to the maximum level that produced a mild tingling sensation without pain (0.8 mA for this patient). taVNS was administered simultaneously with acupuncture for 30 min per session.

### 2.3 Assessment and recording

Patients completed a daily standardized sleep diary documenting bedtime, wake-up time, sleep onset latency, number of nocturnal awakenings, total sleep time, and subjective sleep quality. Sleep efficiency was calculated as (total sleep time/time in bed) × 100%. Outcome measures including PSQI, ISI, HAMA, HAMD-17, CES-D, and HADS-A were assessed at baseline, week 2, week 4 (end of treatment), and week 8 (4-week follow-up).

Outcome measures including PSQI, ISI, HAMA, HAMD-17, CES-D, and HADS-A were assessed at baseline, week 2, week 4 (end of treatment), and week 8 (4-week follow-up).

The intervention lasted 4 weeks, with 3 sessions weekly (12 total sessions). Each session included simultaneous acupuncture and taVNS: acupuncture retained for 60 min, taVNS administered for 30 min. The acupuncture prescription focused on regulating heart and kidney, strengthening spleen and calming spirit.

### 2.4 Clinical course and outcomes

#### 2.4.1 Weeks 1–2 (mid-treatment assessment)

The patient initially felt mild anxiety toward auricular electrical stimulation; intensity was initiated at 0.5 mA and gradually increased to 0.8 mA with explanation of vagal neuromodulation and cardioprotective effects. She reported a calming effect from combined acupuncture and taVNS, with reduced pre-sleep rumination. Sleep diary showed: total sleep time increased from ∼5 h to 6 h; sleep efficiency rose from 73.21% to 76.39%; sleep-onset latency shortened from 50 min to 20 min; nocturnal awakenings decreased from 4 to ∼2 times. Scale scores: PSQI 9, ISI 10, HAMA 18, CES-D 21, HADS-A 9, HAMD-17 9. Palpitations resolved; bowel movements improved.

#### 2.4.2 Weeks 3–4 (post-treatment assessment)

Sleep temporarily worsened (4-5 h/night) during workplace assessment with increased anxiety. Baihui, Sishencong, and Benshen were added to stabilize mental state. A crisis plan was established: fixed wake time, no daytime napping to preserve sleep drive. After the assessment, sleep rapidly recovered. By week 4: sleep-onset latency 20-30 min; nocturnal awakenings with return to sleep within 15 min; total sleep time 7 h; sleep efficiency 87.5%; daily soft bowel movements. Scale scores: PSQI 8, ISI 10, HAMA 14, CES-D 20, HADS-A 8, HAMD-17 7.

#### 2.4.3 Week 8 (4-week follow-up)

No further acupuncture or taVNS was administered. The patient maintained stable sleep using sleep hygiene and self-acupressure. Transient sleep fluctuations occurred only during stress, without severe insomnia. Scale scores: PSQI 8, ISI 5, HAMA 9, HADS-A 7, CES-D 15, HAMD-17 3. Average sleep parameters: total sleep time 7 h, sleep efficiency 82.35%, sleep-onset latency 25 min, ≤1 nocturnal awakening. The patient was satisfied with outcomes, discontinued all sedative supplements, and declined polysomnography.

### 2.5 Summary of outcomes

After 4 weeks of intervention and 4 weeks of follow-up, sleep continuity, daytime function, and emotional state improved significantly. Sleep-onset latency decreased from 45 min to 20 min; total sleep time increased from 5 h to 7 h; sleep efficiency rose from 73.21% to 82.35%. Scale changes: PSQI 14, 9, 8, 8. CES-D 27, 21, 20, 15. HADS-A 8, 9, 8, 7. ISI 13, 10, 10, 5. HAMA 23, 18, 14, 9. HAMD-17 9, 9, 7, 3. No hypnotic medications or adverse events were recorded throughout the study (Table 1; Figure 1).

**Table 1:** Point selection.

**Category**	**Acupuncture Point**	**Meridian**	**Needle Direction**	**Insertion Angle**
Main Points	Shenmen (HT7)	Heart Meridian of Hand-Shaoyin (Yin)	Toward the head	45° oblique
	Shaohai (HT3)	Heart Meridian of Hand-Shaoyin (Yin)	Toward the hand	45° oblique
	Yingu (KI10)	Kidney Meridian of Foot-Shaoyin (Yin)	Toward the head	45° oblique
	Zusanli (ST36)	Stomach Meridian of Foot-Yangming (Yang)	Toward the head	45° oblique
Adjunctive Points (added from Week 3)	Baihui (DU20)	Governor Vessel	Transverse (horizontal)	15° transverse
	Sishencong (EX-HN1)	Extraordinary Point	Transverse (horizontal)	15° transverse

**Table 2:** Clinical outcomes.

Measure	**Baseline**	**Week 2**	**Week 4**	**Week 8**
PSQI total score	14	9	8	8
Sleep quality	2	1	1	1
Sleep latency	2	2	2	2
Sleep duration	3	2	2	1
Sleep efficiency	2	1	0	1
Sleep disorder	2	1	1	1
Sleep medication	0	0	0	0
Daytime dysfunction	3	2	2	2
ISI score	13	10	10	5
CES-D score	27	21	20	15
HAMD-17 score	9	9	7	3
HADS-A score	8	9	8	7
HAMA score	23	18	14	9

**Figure 1:**
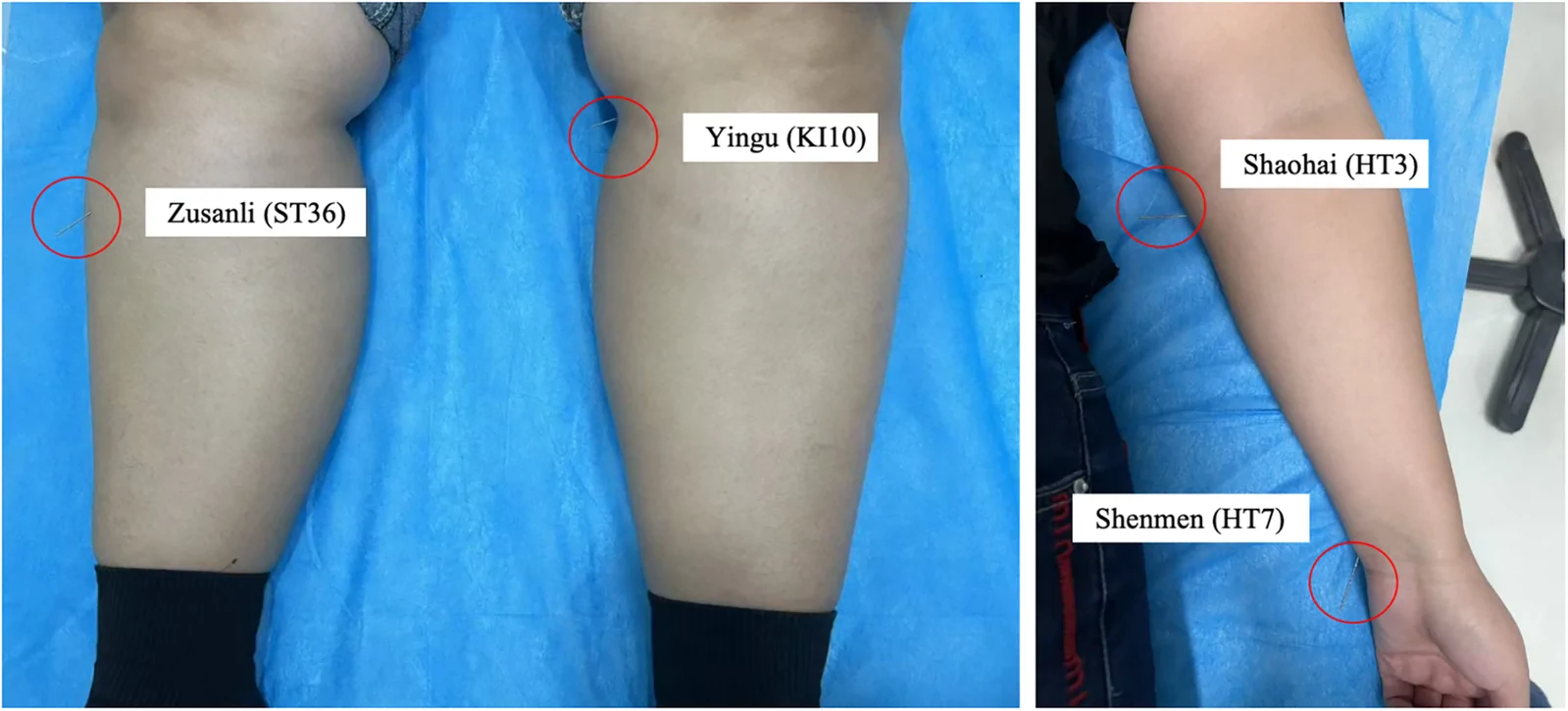
Acupuncture treatment plan (point selection in the afternoon).

**Figure 2:**
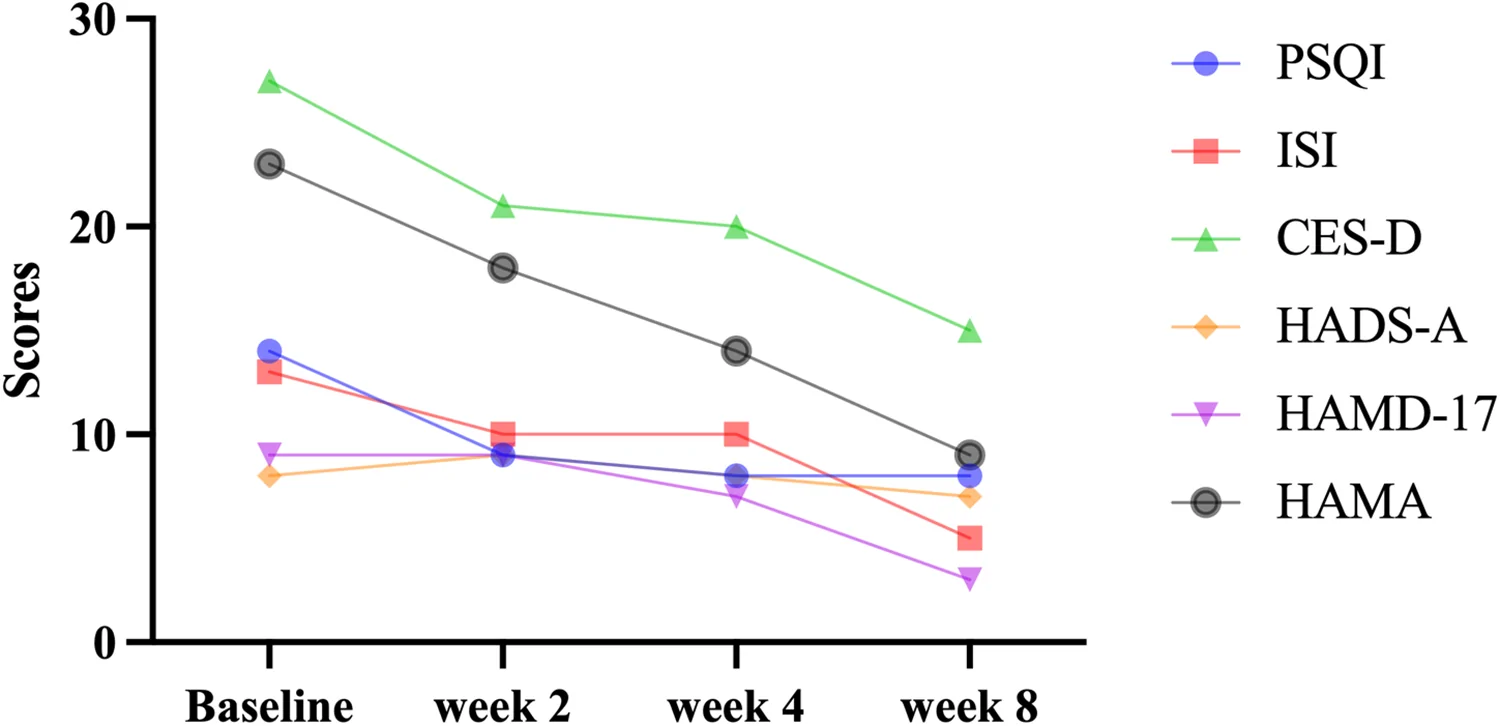
Scale ratings.

## 3 Discussion

This case report presents a promising non-pharmacological treatment outcome in a patient with long-standing postpartum-onset chronic insomnia complicated by persistent anxiety and depressive symptoms. The patient's sleep disturbance persisted for 2 years after delivery and was further aggravated by occupational stress and social isolation, forming a self-sustaining insomnia-emotion cycle evidenced by elevated baseline PSQI, CES-D, and HAMA scores. Given the patient's explicit refusal of pharmacotherapy, combined acupuncture and taVNS was implemented as a standalone mind-body regulatory intervention based on TCM patterns of heart-kidney disharmony and heart-spleen deficiency.

The synergistic intervention targeted both TCM-derived pathological mechanisms and modern neurophysiological hyperarousal. Time-regulated directional acupuncture was applied to tranquilize the mind and harmonize the heart and kidney, consistent with existing evidence supporting acupuncture as an effective treatment for insomnia (25). Consistent with the nature of chronic psychosomatic disorders, objective sleep parameters improved rapidly over four weeks of treatment, while subjective sleep, depressive, and anxiety symptoms resolved more gradually.

Notably, clinical improvements continued to progress during the four-week treatment-free follow-up period, demonstrating a sustained carry-over effect of the combined intervention. This long-term beneficial shift suggests that short-term integrated neuromodulation may disrupt the vicious cycle between insomnia and negative emotion and rebuild adaptive sleep regulation, potentially via neuroplastic modifications in sleep–wake circuitry. Nevertheless, the observed spontaneous improvement cannot be solely attributed to specific therapeutic effects. Confounding variables include ongoing sleep hygiene adherence, self-applied acupressure, natural fluctuation of chronic insomnia, and possible regression to the mean after high-baseline symptom severity.

Compared with hypnotic medications, which often produce transient effects and rebound insomnia upon discontinuation, acupuncture and taVNS may induce durable regulatory changes, supporting the clinical value of short-term intensive intervention followed by self-management. However, the synergistic mechanism underlying the combined protocol remains preliminary. Complementarity between acupuncture and taVNS is inferred from their distinct working pathways, while direct empirical evidence for synergistic interaction is still lacking. In addition, the absence of sham controls and objective sleep monitoring, such as polysomnography or actigraphy, means all efficacy assessments rely primarily on subjective rating scales, limiting the strength of causal inference.

Clinically, this case highlights important practical considerations. Transient sleep rebound triggered by acute stress was successfully managed via flexible acupoint adjustment and cognitive guidance, maintaining treatment compliance. The patient's medical background facilitated accurate symptom self-monitoring and high adherence, which favored treatment response. For vulnerable postpartum individuals with limited social support and high stress, enhancing psychological resilience and self-management capacity is therefore essential for sustained sleep improvement.

The current case possesses unique clinical novelty. Differing from most studies focusing on early postpartum insomnia, this report targets chronic insomnia persisting long after the perinatal period, providing new evidence for late-stage postpartum insomnia management. The medication-free setting further excludes pharmacological interference, enabling pure evaluation of non-pharmacological combined intervention outcomes. Moreover, the integration of time-differentiated directional acupuncture and non-invasive taVNS represents an unreported combinatorial protocol for chronic postpartum insomnia, offering an innovative therapeutic strategy for refractory clinical cases.

Several key limitations must be acknowledged. First, as a single-case study without a control condition, spontaneous remission, placebo effects, and subtle lifestyle influences cannot be fully ruled out. Second, outcomes depend entirely on subjective questionnaires and sleep diaries, lacking objective sleep architecture verification. Third, the patient's high health literacy and good compliance may reduce generalizability to general postpartum populations. Fourth, the short four-week follow-up limits conclusions regarding long-term efficacy stability. Finally, this study cannot dissociate the independent contributions of acupuncture and taVNS, leaving their synergistic mechanism unconfirmed. Despite these constraints, this case supports the safety and clinical potential of combined acupuncture–taVNS therapy for chronic stress-related postpartum insomnia with emotional comorbidity. Future large-sample randomized controlled trials with sham controls, objective sleep recordings, and extended follow-up are warranted to validate and refine this integrated intervention.

## 4 Conclusion

In this patient with chronic postpartum insomnia complicated by anxiety and depression during solo advanced training, 4 weeks of combined acupuncture and taVNS produced significant, sustained improvements in sleep quality, sleep-onset latency, sleep efficiency, and emotional symptoms. The tailored point selection and yin-yang time-based needling targeted heart-kidney disharmony and heart-spleen deficiency, while acupuncture and taVNS synergistically corrected autonomic imbalance. This combined non-pharmacological regimen provides a practical strategy for managing chronic postpartum insomnia during life transitions. However, the current evidence derives from a single-case observation, and placebo effects, natural disease fluctuations, and confounding factors cannot be excluded. The true efficacy of the long-term effects requires verification through large-sample RCTs with active comparators and sham controls. Future studies should further differentiate the independent contributions of acupuncture and taVNS, incorporate objective sleep measures such as PSG, extend follow-up to at least 6 months, and include postpartum populations with diverse health literacy levels to enhance generalizability.

## Data Availability

The original contributions presented in the study are included in the article/Supplementary Material, further inquiries can be directed to the corresponding author.

## References

[1] Morin CM Benca R. Chronic insomnia. Lancet. (2012) 379(9821):1129–41. 10.1016/S0140-6736(11)60750-2 22265700

[2] Roth T. Insomnia: definition, prevalence, etiology, and consequences. J Clin Sleep Med. (2007) 3(5 Suppl):S7–10. 10.5664/jcsm.26929 17824495 PMC1978319

[3] Zhang B Wing YK. Sex differences in insomnia: a meta-analysis. Sleep. (2006) 29(1):85–93. 10.1093/sleep/29.1.85 16453985

[4] Palagini L Cipriani E Caruso V Sharma V Gemignani A Bramante A. Insomnia during the perinatal period and its association with maternal and infant psychopathology: a systematic review and meta-analysis. Curr Psychiatry Rep. (2023) 25(11):617–41. 10.1007/s11920-023-01463-3 37819491

[5] Swanson LM Kalmbach DA Raglan GB O'Brien LM. Perinatal insomnia and mental health: a review of recent literature. Curr Psychiatry Rep. (2020) 22(12):73. 10.1007/s11920-020-01198-5 33104878 PMC9109228

[6] Sivertsen B Hysing M Dørheim SK Eberhard-Gran M. Trajectories of maternal sleep problems before and after childbirth: a longitudinal population-based study. BMC Pregnancy Childbirth. (2015) 15:132. 10.1186/s12884-015-0577-1 26031504 PMC4458335

[7] McBean AL Montgomery-Downs HE. What are postpartum women doing while the rest of the world is asleep? J Sleep Res. (2015) 24(3):270–8. 10.1111/jsr.12265 25431167

[8] Matsumoto K Shinkoda H Kang MJ Seo YJ. Longitudinal study of mothers’ sleep-wake behaviors and circadian time patterns from late pregnancy to postpartum-monitoring of wrist actigraphy and sleep logs. Biol Rhythm Res. (2003) 34(3):265–78. 10.1076/brhm.34.3.265.18812

[9] Quin N Lee JJ Pinnington DM Newman L Manber R Bei B. Differentiating perinatal insomnia disorder and sleep disruption: a longitudinal study from pregnancy to 2 years postpartum. Sleep. (2022) 45(2):zsab293. 10.1093/sleep/zsab293 34989808 PMC9013000

[10] Bei B Pinnington DM Quin N Shen L Blumfield M Wiley JF. Improving perinatal sleep via a scalable cognitive behavioural intervention: findings from a randomised controlled trial from pregnancy to 2 years postpartum. Psychol Med. (2021) 52(15):3650–60. 10.1017/S0033291721001860 PMC8738775 34231450

[11] Furukawa Y Sakata M Yamamoto R Nakajima S Kikuchi S Inoue M. Components and delivery formats of cognitive behavioral therapy for chronic insomnia in adults: a systematic review and component network meta-analysis. JAMA Psychiatry. (2024) 81(4):357–65. 10.1001/jamapsychiatry.2023.5060 38231522 PMC10794978

[12] Siebern AT Suh S Nowakowski S. Non-pharmacological treatment of insomnia. Neurotherapeutics. (2012) 9(4):717–27. 10.1007/s13311-012-0142-9 22935989 PMC3480569

[13] Zhao F-Y Spencer SJ Kennedy GA Zheng Z Conduit R Zhang W-J. Acupuncture for primary insomnia: effectiveness, safety, mechanisms and recommendations for clinical practice. Sleep Med Rev. (2024) 74:101892. 10.1016/j.smrv.2023.101892 38232645

[14] Park HJ Park HJ Chae Y Kim JW Lee H Chung J-H. Effect of acupuncture on hypothalamic-pituitary-adrenal system in maternal separation rats. Cell Mol Neurobiol. (2011) 31(8):1123–7. 10.1007/s10571-011-9718-x 21643998 PMC11498392

[15] Zhang F Zhang X Peng Q Tang L. Electroacupuncture of the cymba concha alleviates p-chlorophenylalanine-induced insomnia in mice. Acupunct Med. (2023) 41(6):345–53. 10.1177/09645284231160193 37081732

[16] Huang A Xiao G Chen Y Hu Z Lee P-H Huang Y. Ziwuliuzhu acupuncture modulates clock mRNA, Bmal1 mRNA and melatonin in insomnia rats. J Acupunct Meridian Stud. (2023) 16(3):109–18. 10.51507/j.jams.2023.16.3.109 37381033

[17] Luckey AM Adcock K Vanneste S. Peripheral nerve stimulation: a neuromodulation-based approach. Neurosci Biobehav Rev. (2023) 149:105180. 10.1016/j.neubiorev.2023.105180 37059406

[18] Yeom JW Kim H Park S Yoon Y Seo JY Cho C-H. Transcutaneous auricular vagus nerve stimulation (taVNS) improves sleep quality in chronic insomnia disorder: a double-blind, randomized, sham-controlled trial. Sleep Med. (2025) 133:106579. 10.1016/j.sleep.2025.106579 40398066

[19] Zou N Zhou Q Zhang Y Xin C Wang Y Claire-Marie R. Transcutaneous auricular vagus nerve stimulation as a novel therapy connecting the central and peripheral systems: a review. Int J Surg. (2024) 110(8):4993–5006. 10.1097/JS9.0000000000001592 38729100 PMC11326027

[20] Zhang S He J-K Meng H Zhao B Zhao Y-N Wang Y. Effects of transcutaneous auricular vagus nerve stimulation on brain functional connectivity of medial prefrontal cortex in patients with primary insomnia. Anat Rec (Hoboken). (2021) 304(11):2426–35. 10.1002/ar.24785 34623769

[21] Shi X Chen L Li W Wang R. Research progress in the prevention and treatment of insomnia with classical prescriptions. J. Cent. South Univ. (Med. Sci.). (2023) 48(10):1494–1505. 10.11817/j.issn.1672-7347.2023.230200 PMC10929891 38432879

[22] Xia N Jiang C Zhou Y Huang Q Hu L Zeng H. A double-blind, randomized, placebo-controlled, single-center clinical trial of jiaotaiwan for the treatment of insomnia caused by disharmony of the heart and kidney. Front Pharmacol. (2022) 13:1011003. 10.3389/fphar.2022.1011003 36408238 PMC9672088

[23] Liu M Zhang Q Yang L. Research advances in the efficacy and mechanism of guipi capsule in reducing insomnia. Evidence-Based Complementary Altern Med. (2024) 2024:1268107–7184. 10.2147/IJGM.S532993

[24] Chen Li Fei X. Effect of acupuncture at Hegu(LI4) on autonomic nerve function in patients with insomnia due to heart-spleen deficiency. J. Clin. Pers. Med. (2024) 3(3):914–923. 10.12677/jcpm.2024.33131

[25] Ang L Lee MS Choi TY. Acupuncture for insomnia. Cochrane Database Syst Rev. (2025) 7(7): CD015974. 10.1002/14651858.CD015974 40626505 PMC12236059

